# Molecular and Genetic Characterization of *Arcobacter* Species Isolated from Chicken Feces and Chicken Giblets from Grenada, West Indies

**DOI:** 10.3390/microorganisms13071495

**Published:** 2025-06-26

**Authors:** Jacqueline Paige Coughlin, Andy Alhassan, Alfred Chikweto, Rohini Roopnarine, Bhumika Sharma

**Affiliations:** Department of Pathobiology, School of Veterinary Medicine, St. George’s University, Grenada, West Indies; jcoughl1@sgu.edu (J.P.C.); aalhass1@sgu.edu (A.A.); achikweto@sgu.edu (A.C.); rroopnarine@sgu.edu (R.R.)

**Keywords:** broiler chicken, *Arcobacter*, polymerase chain reaction (PCR), Enterobacterial Repetitive Intergenic Consensus (ERIC), giblets

## Abstract

This study aimed to isolate and genetically characterize *Arcobacter* species from broiler chickens sampled at three slaughterhouses in Grenada, West Indies. A total of 126 samples—including cloacal swabs, intestinal contents, and meat—from 42 birds were cultured using a chromogenic agar medium. *Arcobacter* spp. were detected in 21.4% (9/42) of the birds. Among the sample types, meat exhibited the highest prevalence at 14.3% (6/42), followed by fecal samples at 7.1% (3/42) and cloacal swabs at 2.4% (1/42). Genus- and species-specific polymerase chain reaction assays on 33 isolates identified five *Arcobacter* species: *A. butzleri*, *A. cryaerophilus*, and *A. skirrowii* (each 18.2%), as well as *A. cibarius* and *A. thereius* (each 6.1%). Genetic diversity was further assessed via Enterobacterial Repetitive Intergenic Consensus–polymerase chain reaction, which revealed 13 distinct genotypic fingerprints forming six clusters, with a high discriminatory power (D = 0.96). This study represents the first documented isolation and molecular characterization of five *Arcobacter* species from broiler chickens in Grenada across multiple sample types. These findings underscore the zoonotic implications of isolating *Arcobacter* spp., particularly in contaminated poultry meat destined for human consumption. The presence of *Arcobacter* spp. in poultry carcasses poses a significant public health concern. To mitigate this public health risk, recommendations include surveillance for the presence of this pathogen in Hazard Analysis and Critical Control Points plans or other tools used to identify pathogens compromising food safety and public health.

## 1. Introduction

The genus *Arcobacter* (synonym: *Aliarcobacter*) [1] comprises Gram-negative bacterial organisms that are non-spore-forming spiral rods and microaerophiles, measuring 0.2–0.9 μm wide and 0.5–3 μm long. *Arcobacter cryaerophilus* was the first species isolated from bovine and pig fetuses in 1977 in Belfast, UK [2]. However, at the time, it was classified within the genus *Campylobacter* and denoted as *Campylobacter cryaerophila*. The *Arcobacter* species were commonly referred to as “campylobacter-like” or “aerotolerant campylobacters” since these resembled *Campylobacter*. However, between 1991 and 1992, many of these “aerotolerant campylobacters” species were reclassified and placed under the new genus *Arcobacter* [3,4]. Since then, the genus *Arcobacter* has undergone multiple revisions and reclassifications [1,5,6,7,8,9]. Currently, there are 33 known species in this genus.

In recent years, *Arcobacter* has emerged as a significant foodborne pathogen, gaining considerable research attention [10]. Over the past decade, *A. butzleri*, *A. cryaerophilus*, and *A. skirrowii* have been linked to human illnesses. Specifically, *A. butzleri* has been associated with gastroenteritis and bacteremia in humans [11,12,13,14,15]. While diarrhea, akin to *Campylobacter* infections, is the primary symptom of *Arcobacter* species infections, *A. butzleri* typically causes persistent, watery diarrhea, in contrast to bloody diarrhea induced by *C. jejuni* [11]. Additional symptoms of *A. butzleri* infections include abdominal cramps, nausea, vomiting, and fever, and in severe cases, hospitalization may be necessary for patients [16]. Consequently, in 2002, the International Commission on Microbiological Specifications for Foods (ICMSF) classified *A. butzleri* as a significant threat to human health [17], likely due to the rising incidence of both individual cases and outbreaks associated with this pathogen [16,18,19,20,21,22]. *Arcobacter butzleri* and *A. cryaerophilus* have also been recognized as potential etiological agents for “Traveler’s Diarrhea” [23,24,25]. The most likely route of transmission of *Arcobacter* species to humans is through the consumption of contaminated or undercooked poultry meat [13,26,27,28].

In the Caribbean region, most people consume poultry meat since it is considered an efficient and cheap source of protein. Across the region, the annual per capita consumption of poultry meat varies from 74.7 kg in Saint Vincent and the Grenadines to 10.9 kg in Haiti, with Grenada at 37.4 kg [29]. Therefore, extensive research has focused on examining poultry meat for the presence of foodborne pathogens in the Caribbean region, with predominant infections attributed to pathogens from the *Campylobacter* and *Salmonella* genera [30,31,32,33,34,35,36,37,38,39]. Conversely, *Arcobacter* species have been documented in poultry and poultry products across many countries [40,41,42,43,44,45,46,47], but their presence has not been established in the Caribbean region, including Grenada. However, there was one report on an incidental finding of *A. butzleri* from pet tortoises in Grenada [48]. *Arcobacter* is categorized as an “emerging foodborne pathogen”; therefore, it is not included on the diagnostic list of known foodborne pathogens [49]. Consequently, the culture protocols primarily focus on detecting other well-established foodborne pathogens such as *Campylobacter*, *Salmonella*, and *Shigella*. Interestingly, other food items of animal origin like beef, pork, shellfish, cheese, and raw milk have been documented to exhibit contamination by *Arcobacter* species; however, poultry and poultry-derived products demonstrate the highest incidence of *Arcobacter* species [50,51,52,53,54].

In light of the above background, this study aimed at isolating and characterizing *Arcobacter* species from broiler chickens from three local farms in Grenada. A commercially available chromogenic agar medium was utilized for the accurate morphological identification of the bacterium. Further molecular characterization of the isolates was performed through the polymerase chain reaction (PCR) technique.

## 2. Materials and Methods

### 2.1. Sample Collection

Sample collection was performed per the approved Institutional Animal Care and Use Committee protocols (IACUC-20007-R) of Saint George’s University (SGU), Grenada, West Indies. Forty-two broiler chickens were sampled from three different slaughter farms in Grenada, West Indies. From each bird, a cloacal sample was collected using a sterile flocked swab (Puritan™ HydraFlock™, Fisher Scientific, Pittsburgh, PA, USA). Each swab was placed in a transport tube containing 3 mL of 1× Phosphate-Buffered Saline (PBS) and maintained in a cold storage box. After slaughter, meat samples were collected from the birds, including breast muscle (*n* = 3), gizzard (*n* = 11), heart (*n* = 9), and liver (*n* = 19). Additionally, fecal samples (*n* = 42), obtained from the intestinal contents, were collected from each bird. These samples were placed in a sterile container and transported on ice to the Microbiology Laboratory of the School of Veterinary Medicine, SGU, along with the swab samples, and processed within 4 h of collection.

### 2.2. Enrichment and Culture Isolation of Arcobacter

Samples in each category were enriched in Houf Broth (HB). Houf Broth was prepared by supplementing 5-fluorouracil (100 mg/L), amphotericin B (5 mg/L), cefoperazone (16 mg/L), novobiocin (32 mg/L), and trimethoprim (64 mg/L) in Arcobacter-Specific Broth (Oxoid, Basingstoke, Hampshire, UK) [55]. Each cloacal swab tip was pressed against the transport tube wall containing 1× PBS to squeeze out the sample. In total, 1 mL of the sample was added to 9 mL of HB. Approximately 1 g of each fecal sample was inoculated in 9 mL of HB and thoroughly mixed by vortexing. For the meat samples, approximately 1 g was placed in a stomacher bag containing 5 mL of 1× PBS. The sample was hand massaged for 1–2 min to expose the entire surface of the meat sample. In total, 1 mL of the sample was inoculated into 9 mL of HB and mixed by vortexing. All the samples were incubated at 30 °C for 48 h. After incubation, the samples were streaked onto Nguyen–Restaino–Juárez (NRJ)-Arcobacter Chromogenic Agar media plates (NRJ-M) (R & F^®^ Products, Downers Grove, IL, USA). The agar plates were incubated aerobically at 30 °C for 48 to 72 h. Presumptive *Arcobacter* was identified by observing the bacterial growth on the NRJ-M plates. On NRJ-M plates, *Arcobacter* species appear as salmon flat to raised colonies, 0.5–1.5 mm in diameter, with or without a clear ring after 72 h at 30 °C under aerobic conditions [56,57]. Such colonies were sub-cultured for purification on another NRJ-M plate and incubated at 30 °C for 48 h. Presumptive *Arcobacter* isolates were subjected to Gram-staining, wet mounting, and biochemical tests.

### 2.3. Nucleic Acid Extraction from the Bacterial Isolates

Genomic Deoxyribonucleic Acid (gDNA) was extracted from the bacterial isolates using the DNeasy Blood & Tissue Kit (Qiagen, Hilden, Germany). The extraction protocols provided by the kit manufacturers were followed. American-Type Culture Collection (ATCC; Manassas, VA, USA)-derived *Arcobacter* reference strains for *A. butzleri* (ATCC 49616), *A. cryaerophilus* (ATCC 43158), *A. skirrowii* (ATCC 51400), RM 4473 (*A. butzleri*), and RM 4598 (*A. cryaerophilus*) were used as positive controls. Genomic DNA from these reference strains was extracted separately using the DNeasy Blood & Tissue Kit (Qiagen, Hilden, Germany). The purity and concentrations of the gDNA were detected using the NanoDrop™ 2000 Spectrophotometer (Eppendorf, Hauppauge, NY, USA). The gDNA samples were then stored at −20 °C until further molecular and genetic characterizations were performed.

### 2.4. Molecular Characterization of the Arcobacter Species

The gDNA samples were subjected to *Arcobacter* genus-specific single-plex PCR. This PCR amplified a 16S rRNA gene fragment universally found in all *Arcobacter* species [58]. The samples that were PCR-positive for the genus-specific protocol were further subjected to a multiplex PCR that simultaneously amplified gene fragments from five *Arcobacter* species: *butzleri*, *skirrowii*, *thereius*, *cibarius*, and *cryaerophilus* [59]. Table 1 shows the sequences and origins of all primers used for gene amplification.

The PCR master mix preparation and cycling conditions for the single-plex assay were as follows: A total of 25 µL of reaction mixture was prepared that contained a final concentration of 1× Platinum PCR High Fidelity Supermix, 0.4 µM of the forward and reverse primers, and 1 μL (~10 ng to 30 ng) of DNA template. Samples were initially heated for 2 min at 94 °C, followed by 35 amplification cycles of 30 s. at 94 °C (denaturation), 30 s. at 56 °C (primer annealing), and 1 min at 68 °C (primer extension). A final elongation step (68 °C for 5 min) followed the final amplification cycle.

The PCR master mix preparation and cycling conditions for the multiplex assay were as follows: A total of 25 µL of reaction mixture was prepared that contained a final concentration of 1× High Fidelity PCR buffer, 0.2 mM of dNTPs, 1.5 mM of MgSO_4_, 0.5 µM of the forward and reverse primers for the five *Arcobacter* species, 1.5 U of the Taq DNA Polymerase, and 1 μL (~10 ng to 30 ng) of DNA template. Samples were initially heated for 3 min at 94 °C followed by 35 amplification cycles of 45 s. at 94 °C (denaturation), 45 s. at 58 °C (primer annealing), and 2 min at 68 °C (primer extension). A final elongation step (68 °C for 5 min) followed the final amplification cycle.

### 2.5. Sequencing of the Samples 

For direct sequencing purposes, the positive samples were subjected to PCRs using the primers listed in Table 2 to amplify gene sequences for *Arcobacter* species. Twenty-five microliters of the PCR products were subjected to electrophoresis with a 1.5% agarose gel, stained with ethidium bromide, and photographed under a gel documentation system (LabNet International Inc., Edison, NJ, USA). Positively identified amplicons were purified using the QIAquick Gel Extract Kit (Qiagen, Hilden, Germany) following the manufacturer’s instructions and sent for direct sequencing to the sequencing facility provided by Molecular Cloning Laboratories (South San Francisco, CA, USA). The sequencing histograms were processed and compared to the GenBank^®^ sequence database using the Standard Nucleotide Basic Local Alignment Search Tool (BLASTN) (https://blast.ncbi.nlm.nih.gov/Blast.cgi; accessed on 23 June 2025) provided by the National Center for Biotechnology Information (NCBI). The comparison was restricted to the RefSeq Reference Genome Database, specifically filtered to include only sequences belonging to the *Arcobacter* Group (taxonomic ID: 2808963).

### 2.6. Genetic Characterization of the Arcobacter Species

The *Arcobacter* species identified through the molecular assays and sequencing were subjected to genetic profiling. The Enterobacterial Repetitive Intergenic Consensus–PCR (ERIC-PCR) assay was performed on all *Arcobacter* species for the genetic fingerprinting of the bacterial genomes. The primers designed by Versalovic et al. [61] were used to perform the PCR: ERIC1R (5′-ATGTAAGCTCCTGGGGATTCAC-3′) and ERIC2 (5′-AAGTAAGTGACTGGGGTGAGCG-3′). These primers were used in a PCR protocol described by Houf et al. [62]. Briefly, a 50 μL reaction mixture containing a final concentration of 1× PCR reaction buffer, 0.5 μM of the forward primer and reverse primer, 0.2 mM of the dNTPs, 4 mM of MgCl_2_, 5 U of Platinum Taq DNA polymerase, and ~5–25 ng/μL of template DNA was used for each reaction. Nuclease-free water was used as a negative control, and the *Arcobacter* reference strains from ATCC were used as positive controls. The cycling conditions were as follows: initial denaturation at 94 °C for 5 min, followed by 40 cycles of 1 min of denaturation at 94 °C, 1 min of annealing at 25 °C, and 2 min of extension at 72 °C, and a final 5 min extension at 72 °C after the last cycle.

Ten microliters of the ERIC-PCR products were size-separated by electrophoresis in ethidium bromide-stained 1.5% agarose gels with 1× Tris-acetate-EDTA buffer, run for 2.5 h at 100 Volts. The DNA profiles were visualized under a UV transilluminator and photographed using a gel documentation system (LabNet International Inc., Edison, New Jersey, USA). DNA patterns that differ in one or more DNA fragments were considered patterns that represent different types. To interpret the profiles of the isolates, the GelJ software program (version 2.0) [63] was used. GelJ uses the gel picture to generate a computer-based DNA band clustering matrix. Based on the presence or absence of a band, a binary matrix is marked with 1 (or +) or 0 (or −), respectively. This band clustering matrix is used to compute a similarity matrix using the Dice coefficient [64], which expresses the similarity level between two DNA patterns. Based on the similarity matrix, a dendrogram was constructed using the unweighted pair group linkage analysis method (UPGAM). The numerical index of discrimination for ERIC-PCR was calculated by Simpson’s index of diversity [65] using the following formula:

(1) $$D=1-\frac{1}{N\left(N-1\right)}\;\sum_{j=1}^{S}\;n_{j}\;\left(n_{j}-1\right)$$

where *D* = index of discriminatory power; *N* = the total number of strains in the sample population; *n_j_* = the number of strains belonging to the *j*th type; and *s* = the total number of types defined.

## 3. Results

### 3.1. Bacterial Culture

**Figure 1:**
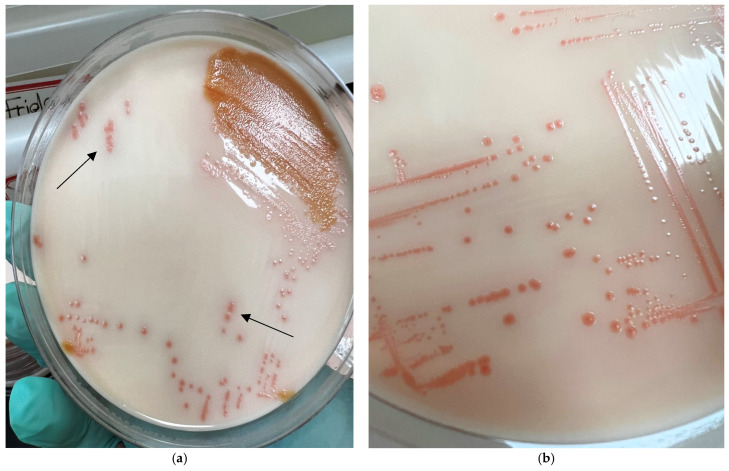
Culture plates from sample M77 growing on the NRJ-chromogenic agar medium. (**a**) Mixed colonies (arrows showing salmon-pink colonies of *Arcobacter*); (**b**) pure isolate of *Arcobacter*.

Culture protocols revealed that 21.4% of birds (9/42) had *Arcobacter* species in their fecal, giblet, and/or cloacal samples. Specifically, three birds had *Arcobacter* in fecal samples, five in giblet samples, and one in both giblet and cloacal swab samples. Out of 126 total samples, meat samples exhibited the highest prevalence of *Arcobacter* spp., with an isolation rate of 14.3% (6/42). Within the meat category, no *Arcobacter* spp. were cultured from breast (0/3) or gizzard (0/11) samples, while heart and liver samples showed isolation rates of 22.2% (2/9) and 21.1% (4/19), respectively. Fecal samples had a lower isolation rate of 7.1% (3/42), and *Arcobacter* spp. were detected in only 2.4% (1/42) of cloacal swab samples. The overall prevalence of *Arcobacter* spp. across all sample types was 7.9% (10/126) (Table 3). Thirty-three isolates were sub-cultured from these ten samples, and on NRJ-M plates, *Arcobacter* colonies appeared salmon-colored, 0.5–1.5 mm in diameter, and flat to raised, with or without a clear ring after 72 h at 30 °C under aerobic conditions (Figure 1). Gram-staining, wet mounting, and microscopy for these isolates showed that they were Gram-negative, curved rods with corkscrew motility. Biochemical tests revealed that the isolates were oxidase-positive, catalase-positive, and Hippurate hydrolysis-negative.

### 3.2. Molecular Characterization and Sequence Analysis of the Isolates

**Figure 2:**
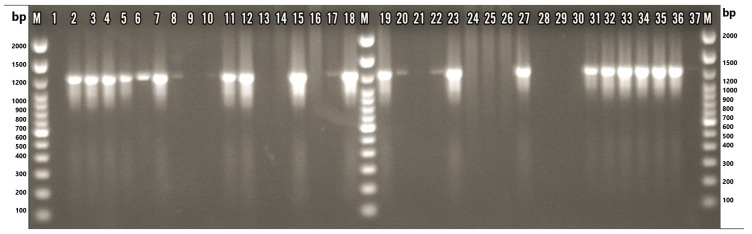
Gel electrophoresis image of samples tested with *Arcobacter* genus-specific PCR. M: TrackIt™ 100 bp DNA Ladder (Thermo Fisher Scientific, Waltham, MA, USA); 1: nuclease-free water; 2: ATCC 49616 (*A. butzleri*); 3: ATCC 43158 (*A. cryaerophilus*); 4: ATCC 51400 (*A. skirrowii*); 5: F45.1; 6: F45.2; 7: F87.1; 8: F87.2; 9: F87.4; 10: F87.3; 11: F88.1; 12: M77.1; 13: M77.2; 14: M77.3; 15: M80.1; 16: M80.2; 17: M80.3; 18: M84.1; 19: M84.2; 20: M84.3; 21: M84.4; 22: M84.5; 23: M86.1; 24: M86.2; 25: M86.3; 26: M86.4; 27: M89.1; 28: M89.2; 29: M89.3; 30: M89.4; 31: M101.1; 32: M101.2; 33: M101.3; 34: M101.4; 35: S84.1; 36: S84.2; 37: S84.3. F: chicken feces; M: chicken meat; S: chicken cloacal swab.

A genus-specific PCR assay targeting the 16S rRNA gene was performed on 33 presumptive *Arcobacter* isolates. Amplification of the expected 1223 bp fragment was observed in 22 isolates (66.7%), thereby confirming their classification within the *Arcobacter* genus (Figure 2). Distribution analysis of the confirmed isolates by sample type showed that 13 isolates (39.4%) originated from giblet samples, 10 (30.3%) from the liver, and three (9.1%) from heart tissue. Additionally, six isolates (18.2%) were obtained from fecal samples, and three (9.1%) from cloacal swabs (Table 3).

**Figure 3:**
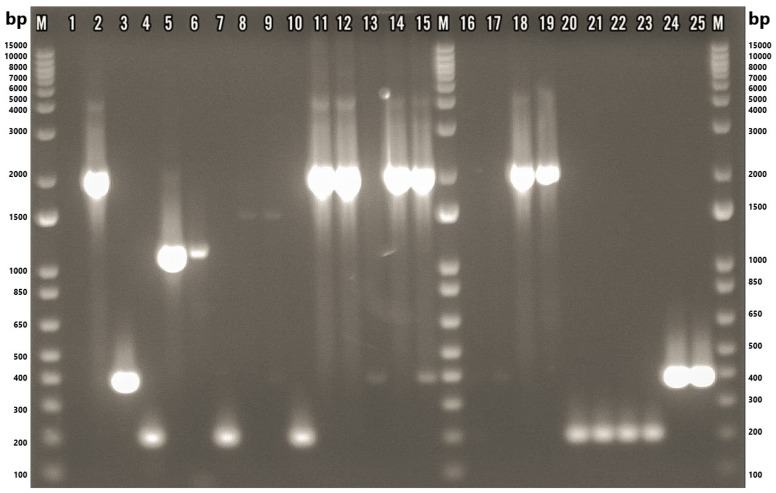
Gel-electrophoresis image of samples tested via multiplex PCR. M: 1 Kb Plus DNA Ladder (Thermo Fisher Scientific, Waltham, MA, USA); 1: nuclease-free water; 2: ATCC 49616 (*A. butzleri*); 3: ATCC 43158 (*A. cryaerophilus*); 4: ATCC 51400 (*A. skirrowii*); 5: F45.1; 6: F45.2; 7: F87.1; 8: F87.2; 9: F87.3; 10: F88.1; 11: M77.1; 12: M80.1; 13: M80.3; 14: M84.1; 15: M84.2; 16: M84.3; 17: M84.5; 18: M86.1; 19: M89.1; 20: M101.1; 21: M101.2; 22: M101.3; 23: M101.4; 24: S84.1; 25: S84.2. F: chicken feces; M: chicken meat; S: chicken cloacal swab.

Species-level identification of the confirmed isolates was subsequently conducted using a multiplex PCR assay. Of the 22 *Arcobacter*-positive isolates, 17 (77.3%) yielded a single species-specific amplicon, indicating mono-species infections. Conversely, two isolates (9.1%) produced multiple bands, suggestive of mixed-species infections. Specifically, isolate F87.3 amplified fragments corresponding to both *A. thereius* (~1590 bp) and *A. cryaerophilus* (~395 bp), while isolate M84.2 showed bands indicative of *A. butzleri* (~2061 bp) and *A. cryaerophilus* (~395 bp) (Figure 3). Two isolates, M84.3 and S84.3 (9.1%), could not be identified at the species level despite sequencing the 16S rRNA gene fragment using the primer pair ArcoI and ArcoII. The resulting chromatograms exhibited poor resolution, which hindered accurate species-level identification. It should be noted that sample S84.3 was only subjected to a genus-specific PCR yielding an extremely faint band and therefore was not subjected to the multiplex PCR.

It is interesting to note that isolate F87.2 exhibited faint amplification products in both the genus- and species-specific PCR assays, which initially limited the ability to clearly identify the constituent species. Sequencing attempts using species-specific primers were unsuccessful due to the low band intensity. However, subsequent sequencing using genus-specific primers successfully identified *Arcobacter cryaerophilus*, and the sequence was deposited in GenBank (accession number PP937576). While the multiplex PCR (Figure 3) did not display a clear band corresponding to *A. cryaerophilus*, the genus-specific PCR (Figure 2) and sequencing results provided confirmatory evidence of its presence. Furthermore, the multiplex PCR revealed a distinct band for *A. thereius* in the same sample. Taken together, these molecular and sequencing results support that sample F87.2 contained both *A. thereius* and *A. cryaerophilus*, and it has, therefore, been classified as a mixed *Arcobacter* isolate.

Analysis of the species distribution by sample type (Table 3) revealed that giblet samples (liver and heart) harbored three *Arcobacter* species, with *A. butzleri* being the most prevalent (15.1%), followed by *A. skirrowii* (12.1%) and *A. cryaerophilus* (6.1%). Fecal samples contained four *Arcobacter* species—*A. cibarius*, *A. cryaerophilus*, *A. thereius*, and *A. skirrowii*—each at a frequency of 6.1%. The cloacal swab samples exclusively yielded *A. skirrowii* (6.1%).

Overall, *A. skirrowii* was the most frequently isolated species (18.2%), predominantly from liver (12.1%) and fecal (6.1%) samples. *A. butzleri* was detected in 15.1% of samples, exclusively in giblet tissue. *A. cryaerophilus* was recovered from both giblet and cloacal swab samples (12.1%), while *A. cibarius* was found only in fecal samples (6.1%).

### 3.3. Sequencing

Of the 20 isolates that tested positive via the multiplex PCR assay, 18 were sequenced using the primers listed in Table 2. Table 4 illustrates a further sample-wise breakdown of these isolates and the species identified via multiplex PCR and sequencing. The BLASTN (https://blast.ncbi.nlm.nih.gov/Blast.cgi; accessed on 23 June 2025) analyses of the amplified gene sequences from these 18 isolates showed between 96 and 100% similarity with the described matches listed in Table 5. These sequences were subsequently deposited into the NCBI GenBank^®^ ([Internet]. Bethesda (MD): National Library of Medicine (US), National Center for Biotechnology Information; [1982]—[cited 23 June 2024]. Available from: https://www.ncbi.nlm.nih.gov/nucleotide/) and were assigned the accession numbers provided in Table 4. Notably, *A. thereius* amplified from samples F87.2 and F87.3 and *A. cryaerophilus* amplified from samples M80.3, M84.2, and M84.5 were not confirmed by direct sequencing since the band intensities were very low.

### 3.4. Genetic Characterization of the Arcobacter Species

**Figure 4:**
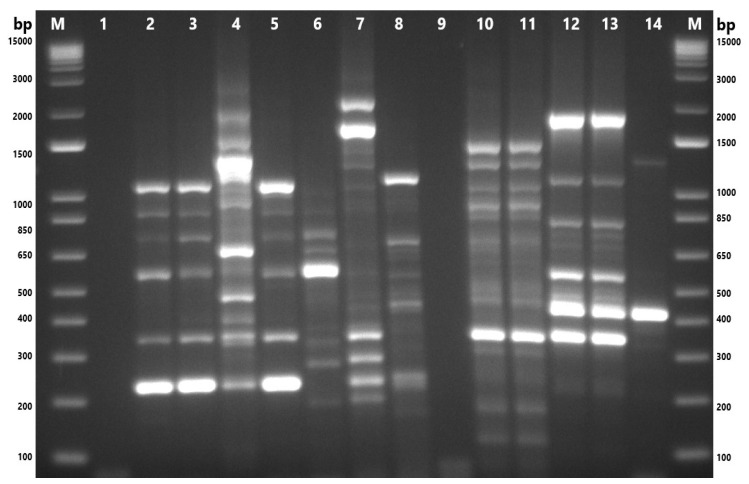
Gel-electrophoresis analysis of Enterobacterial Repetitive Intergenic Consensus–polymerase chain reaction (ERIC-PCR) fingerprinting of *A. butzleri* and *A. cryaerophilus* isolates. M: 1 Kb Plus DNA Ladder (Thermo Fisher Scientific, Waltham, MA, USA); 1and 9: nuclease-free water; 2: M77.1; 3: M80.1; 4: M84.1; 5: M84.2; 6: M86.1; 7: M89.1; 8: ATCC 49616 (*A. butzleri*); 10: F87.2; 11: 87.3; 12: S84.1; 13: S84.2; 14: ATCC 43158 (*A. cryaerophilus*). F: chicken feces; M: chicken meat; S: chicken cloacal swab.

**Figure 5:**
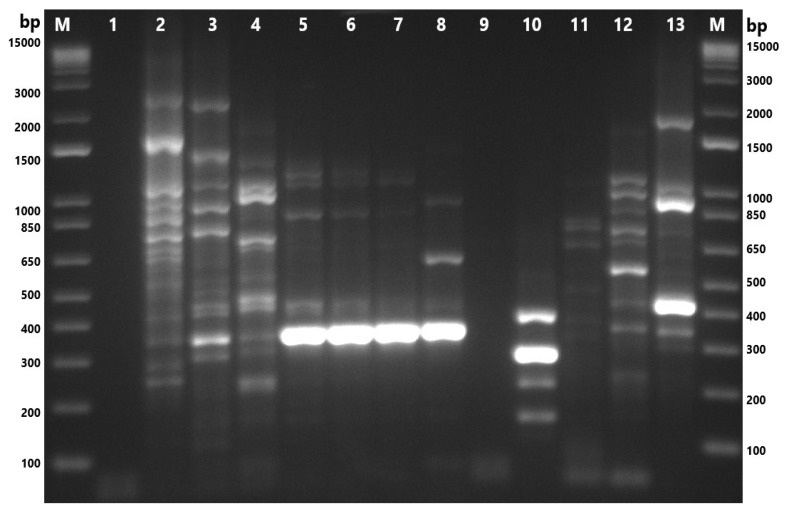
Gel-electrophoresis analysis of Enterobacterial Repetitive Intergenic Consensus–polymerase chain reaction (ERIC-PCR) fingerprinting of *A. skirrowii* and *A. cibarius* isolates. M: 1 Kb Plus DNA Ladder (Thermo Fisher Scientific, Waltham, MA, USA); 1,9: nuclease-free water; 2: F87.1; 3: F88.1; 4: M101.1; 5: M101.2; 6: M101.3; 7: M101.4; 8: ATCC 51400 (*A. skirrowii*); 10: F45.2; 11: F45.1; 12: RM 4473 *(A. butzleri*); 13: RM 4598 (*A. cryaerophilus*). F: chicken feces; M: chicken meat; S: chicken cloacal swab.

**Figure 6:**
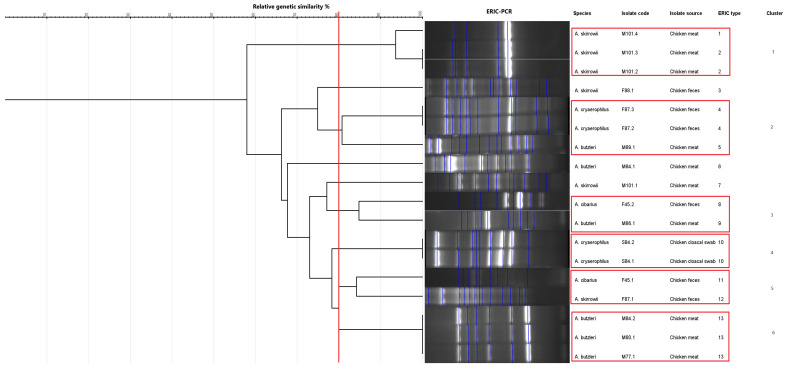
Dendrogram generated from the Enterobacterial Repetitive Intergenic Consensus–polymerase chain reaction (ERIC-PCR) fingerprinting using the GelJ program. The dendrogram was generated by combining the gels of the two ERIC-PCRs (Figure 4 and Figure 5). The red vertical line represents the 80% similarity threshold used for clustering the isolates. Red frames denote groups of isolates that clustered together based on this cut-off.

The genetic characterization of the 18 isolates was performed using ERIC-PCR, which generated banding patterns as shown in Figure 4 and Figure 5. The isolates exhibited 4–13 distinct bands, ranging from ~150 bp to ~2500 bp. These gels and the banding patterns were further analyzed using a software program called GelJ to create a dendrogram, which grouped the isolates into different “ERIC-Types” and “clusters,” providing information on the DNA fingerprints of these isolates (Figure 6). The dendrogram was constructed using the unweighted pair group linkage analysis method (UPGMA), with the cutoff value for clustering the isolates set at 80%. Applying this cutoff value, the isolates were clustered into six groups (each cluster containing 2–3 isolates), resulting in 13 ERIC-Types based on the banding patterns (Figure 6). The discriminatory power, calculated using Simpson’s Index of Diversity, was 0.96.

## 4. Discussion

This is the first study conducted in Grenada, West Indies, to isolate and molecularly characterize *Arcobacter* species from chicken giblets and fecal samples. This study has established the presence of *Arcobacter* species in chickens from Grenada and emphasized the emerging status of *Arcobacter* as a foodborne pathogen. Since *Arcobacters* are closely related to *Campylobacters*, the latter being well-established as foodborne pathogens, the majority of the culture protocols available utilize enrichment media and temperature conditions that favor the growth of *Campylobacters* more than *Arcobacters* [66,67,68]. Although some *Arcobacter*-specific broths and enrichment media are commercially available, these products do not have high sensitivity for isolating *Arcobacters* from field samples (like meat and feces), as the growth of *Arcobacters* is suppressed due to the overgrowth of other species present in samples [69,70]. Therefore, we utilized a commercially available NRJ-chromogenic agar medium that allowed for the easy differentiation of *Arcobacters* based on the salmon-pink color of these colonies, even when present with other bacterial colonies (Figure 1a). Another advantage of using this chromogenic agar medium is that it does not support the growth of other related bacteria like *Campylobacter* and *Helicobacter* due to its composition [56,57,71].

In this study, *Arcobacter* species were cultured from 21% (9/42) of the birds, and all three sample types tested, the highest being the giblet samples (*n* = 6), followed by feces (*n* = 3) and a cloacal swab (*n* = 1). Five *Arcobacter* species were isolated from these samples. The six giblet samples harbored *A. butzleri*, *A. cryaerophilus*, and *A. skirrowii*. The three fecal samples harbored *A. cibarius*, *A. cryaerophilus*, *A. skirrowii*, and *A. thereius*, and one cloacal swab sample harbored *A. cryaerophilus* only. *Arcobacter butzleri* was predominantly present in giblet samples only. *Arcobacter skirrowii* was isolated from giblets and fecal samples. *Arcobacter cibarius* and *A. thereius* were only isolated from feces, and *A. cryaerophilus* was isolated from all three sample types. Of the 33 presumptive *Arcobacter* isolates tested via generic PCR, 22 (66.7%) belonged to the genus *Arcobacter.* Of these 22 isolates subjected to *Arcobacter* species-specific multiplex PCR, 20 isolates (91%) were successfully speciated. Among these, 17 isolates yielded amplification products corresponding to a single *Arcobacter* species (indicating pure isolates). However, three isolates showed amplification bands corresponding to two different *Arcobacter* species, indicating the presence of mixed cultures. This could potentially be attributed to manual error during colony selection, such as inadvertently picking more than one colony. Sanger sequencing of 18 multiplex PCR-positive isolates further confirmed the *Arcobacter* species, which were deposited in the NCBI GenBank database (Table 5).

Research on *Arcobacter* spp. within the Caribbean region remains limited. To date, only three published studies from Costa Rica—a Central American country geographically and ecologically proximal to the Caribbean—have reported on the isolation of *Arcobacter* from poultry products and retail meat sources [40,72,73]. These studies documented *Arcobacter* isolation rates ranging from 20% to 25%, which closely parallels the 21% isolation rate observed in the present study. In the Costa Rican investigations, *Arcobacter* spp. were recovered from various sample types, including poultry birds, retail meat, and fecal content. Among the isolated species, *A. butzleri* was the most prevalent, particularly in chicken meat, with detection rates between 30% and 60%. This was followed by *A. cryaerophilus* (19%) and *A. skirrowii* (4.3%). Comparative data from other geographic regions, such as Turkey, support the high prevalence of *Arcobacter* in poultry-derived products. Two studies, conducted in Turkey [74,75], which analyzed edible giblets and other meats from chickens, bovines, and lambs, reported that chicken giblet samples had the highest contamination levels (23.3% and 62%). In both studies, *A. butzleri* was the most frequently isolated species, with detection frequencies of 60.7% and 67.7%. Similarly, an Egyptian study [76] that focused on edible chicken giblets (livers, gizzards, and fillets) identified liver samples as having the highest contamination rate (30%), followed by fillets (22.5%) and gizzards (20%). In concordance with these findings, our study revealed that giblet samples exhibited the highest *Arcobacter* isolation rate (39.4%) compared to fecal samples (18.2%) and cloacal swabs (9.1%). Across all sample types, the overall isolation frequencies for *A. butzleri*, *A. cryaerophilus*, and *A. skirrowii* (both individually and in combination) were equal at 18.2%, followed by *A. cibarius* and *A. thereius* (6.1%). Notably, *A. butzleri* was isolated exclusively from giblet samples, at the highest individual frequency (15.1%), and was absent from fecal and cloacal swab samples. The observed differences in *Arcobacter* isolation rates across studies may be attributed to several factors, including geographic variability, seasonal influences, hygiene practices during animal product processing, sample size disparities, and methodological differences in bacterial isolation and detection [55,77,78].

Enterobacterial Repetitive Intergenic Consensus–polymerase chain reaction-based genotyping resulted in bands ranging between 4 and 13 (~150 bp to ~2500 bp); all 18 isolates were typable (100%), and a total of 13 ERIC-types and six clusters were observed. It was observed that isolates originating from the same bird or the same sample type clustered together (Figure 6). The ERIC-PCR discriminatory power, calculated using Simpson’s Index of Diversity, was 0.96, indicating ERIC-PCR to be a highly desirable genotyping method since discriminatory powers above 0.90 are considered highly significant [65].

Research shows that the intestinal tracts of live chickens sporadically harbor *Arcobacter* species. As an example, Schonknecht et al. (2020) [47] analyzed intestinal content from 157 samples collected at four stages of poultry slaughter. The authors (47) found that *Arcobacter* spp. are rarely isolated in the intestinal contents after bleeding (6.2%) and have not been isolated after scalding. However, detection increased significantly after defeathering (62%), peaking post-evisceration (90.6%). These findings indicate that the bacteria are not prominent colonizers of the chicken gut during poultry processing. Schonknecht et al. (2020) [47] also reported that the plucking fingers used for defeathering had high levels of *Arcobacter* contamination, suggesting that equipment surfaces serve as reservoirs for *Arcobacter* spp., leading to the cross-contamination of carcasses during processing [79]. These findings are supported by a more recent study [80]. Botta et al. (2024) [80] found a 31% prevalence of *Arcobacter* spp. in a poultry abattoir plucking sector, even after cleaning and sanitizing procedures. The study highlighted the persistence of *Arcobacter* spp. in specific environmental niches within the slaughterhouse, emphasizing that the lack of adequate sanitation and disinfection of slaughter equipment presents a risk of contamination of meat at the poultry plant. Although the present study did not focus on determining the primary sources of contamination in the analyzed sample types, the results support that *Arcobacter* colonizes the chicken gut occasionally and that the meat samples become contaminated during the slaughter process, most likely due to the environmental contamination of the equipment used for slaughter purposes. This “farm-to-fork” contamination route is further exacerbated by the physiological traits of *Arcobacter*, such as its resistance to cold temperatures [81] and aerotolerance, which can increase the probability of final contamination and persistence on broiler carcasses at the retail level [82].

From a public health perspective, the consumption of undercooked or raw poultry products, including their byproducts, presents a significant risk for foodborne illness from contaminated poultry meat. Poultry and related products have been identified as reservoirs for enteropathogenic microorganisms capable of causing gastrointestinal diseases in humans, particularly when proper cooking and hygienic handling practices are not followed [83,84]. Among these pathogens, *Arcobacter butzleri*, *A. cryaerophilus*, and *A. skirrowii* have been associated with human gastrointestinal disorders, such as diarrhea, and in more severe cases, bacteremia [15,28,85,86,87,88]. These species have been isolated from various food sources, including poultry giblets, underscoring their zoonotic potential [74,75]. In light of these findings, it is critical to inform consumers about the health risks associated with the consumption of contaminated chicken products, including the giblets. Preventive strategies should emphasize stringent hygienic handling and thorough cooking of these products to inactivate pathogenic organisms. Public health campaigns should prioritize education and awareness, particularly in regions where the consumption of giblets is common, to reduce the incidence of *Arcobacter*-associated infections. The documented presence of *A. butzleri*, *A. cryaerophilus*, and *A. skirrowii* in chicken giblet samples represents a noteworthy public health concern, especially considering the widespread use of giblets in Caribbean cuisine [89].

## 5. Conclusions

The findings of this study establish the presence of five *Arcobacter* spp. (*A. butzleri*, *A. cryaerophilus*, *A. cibarius*, *A. skirrowii*, and *A. thereius*) in chicken meat and feces from the Caribbean region of Grenada, West Indies. The successful isolation and genetic characterization of five *Arcobacter* species from broiler chickens in Grenada marks a significant advancement in understanding the presence and diversity of this emerging zoonotic pathogen in the region. The prevalence of *Arcobacter* in meat samples underscores the potential for foodborne transmission, emphasizing the need for improved biosecurity measures during poultry processing. Given the documented zoonotic potential of these species, particularly *A. butzleri* and *A. cryaerophilus*, this study highlights an urgent public health concern. Routine surveillance and strict hygienic practices are imperative to mitigate the risk of transmission to humans and to ensure food safety within both local and international markets.

## Figures and Tables

**Table 1:** Oligonucleotide primers used to characterize the samples molecularly.

PCR Assay	Primer Name and Sequence (5′–3′)	Target Gene	Fragment Size (bp)	Target Species	Reference
Single-plex	Arco I: AGAGATTAGCCTGTATTGTAT Arco II: TAGCATCCCCGCTTCGAATGA	16S rRNA	1223	*Arcobacter* spp.	[58]
Multiplex	Arco F: GCTAGAGGAAGAGAAATCAA	23S rRNA		*Arcobacter* spp.	[59]
	But R: TCCTGATACAAGATAATTGTACG	23S rRNA	2061	*A. butzleri*	[59]
	TherR: GCAACCTCTTTGGCTTACGAA	23S rRNA	1590	*A. thereius*	[59]
	CibR: CGAACAGGATTCTCACCTGT	23S rRNA	1125	*A. cibarius*	[59]
	SkiR: TCAGGATACCATTAAAGTTATTGATG	23S rRNA	198	*A. skirrowii*	[59]
	GyrasF: AGAACATCACTAAATGAGTTCTCT GyrasR: CCAACAATATTTCCAGTYTTTGGT	gyrA	395	*A. cryaerophilus*	[59]

**Table 2:** Oligonucleotide primers used for direct sequencing.

Bacterial Species	Primer Name and Sequence (5′–3′)	Target Gene	Fragment Size (bp)	Reference
*A. butzleri*	Arco I: AGAGATTAGCCTGTATTGTAT Arco II: TAGCATCCCCGCTTCGAATGA	16S rRNA	1223	[58]
*A. cibarius*	Arco F: GCTAGAGGAAGAGAAATCAA CibR: CGAACAGGATTCTCACCTGT	23S rRNA	1125	[59]
*A. cryaerophilus*	GyrasF: AGAACATCACTAAATGAGTTCTCT GyrasR: CCAACAATATTTCCAGTYTTTGGT	gyrA	395	[59]
*A. skirrowii*	Arco16S: CGTATTCACCGTAGCATAGC Skir16S: GGCGATTTACTGGAACACA	16S rDNA	641	[60]

**Table 3:** Occurrence of *Arcobacter* spp. in meat, cloacal swab, and feces samples and isolates.

Sample Type	Number of Samples Collected	Number of Samples Positive via Culture (%)	Number of Isolates Positive/Isolates Tested via PCR Assays (%)							
			*Arcobacter* spp.	*butzleri*	*cryaerophilus*	*skirrowii*	*cibarius*	Mix of *butzleri* and *cryaerophilus*	Mix of *cryaerophilus* and *thereius*	Undifferentiated *Arcobacter*
Breast	3	-	-	-	-	-	-	-	-	-
Gizzard	11	-	-	-	-	-	-	-	-	-
Heart	9	2 (22.2)	3/33 (9.1)	2/33 (6.1)	1/33 (3)	-	-	-	-	-
Liver	19	4 (21)	10/33 (30.3)	3/33 (9.1)	1/33 (3)	4/33 (12.1)	-	1/33 (3)	-	1/33 (3)
Subtotal	42	6 (14.3)	13/33 (39.4)	5/33 (15.1)	2/33 (6.1)	4/33 (12.1)	-	1/33 (3)	-	1/33 (3)
Cloacal swabs	42	1 (2.4)	3/33 (9.1)	-	2/33 (6.1)	-	-	-	-	1/33 (3)
Feces	42	3 (7.1)	6/33 (18.2)	-	-	2/33 (6.1)	2/33 (6.1)	-	2/33 (6.1)	-
Total	126	10 (7.9)	22/33 (66.7)	5/33 (15.1)	4/33 (12.1)	6/33 (18.2)	2/33 (6.1)	1/33 (3)	2/33 (6.1)	2/33 (6.1)

**Table 4:** A sample-wise breakdown of the *Arcobacter* isolates and species identified via the multiplex PCR assay and sequencing.

Bird #	Sample #	Isolate #	Species Identified via Multiplex PCR	Confirmed via Sequencing
45	F45	F45.1	*Arcobacter cibarius*	Yes
		F45.2	*Arcobacter cibarius*	Yes
77	M77 (heart)	M77.1	*Arcobacter butzleri*	Yes
80	M80 (heart)	M80.1	*Arcobacter butzleri*	Yes
		M80.3	*Arcobacter cryaerophilus*	No
84	M84 (liver)	M84.1	*Arcobacter butzleri*	Yes
		M84.2	A mix of *Arcobacter butzleri* and *Arcobacter cryaerophilus*	Yes (*A. butzleri* only)
		M84.5	*Arcobacter cryaerophilus*	No
	S84	S84.1	*Arcobacter cryaerophilus*	Yes
		S84.2	*Arcobacter cryaerophilus*	Yes
86	M86 (liver)	M86.1	*Arcobacter butzleri*	Yes
87	F87	F87.1	*Arcobacter skirrowii*	Yes
		F87.2	A mix of *Arcobacter thereius* and *Arcobacter cryaerophilus*	Yes (*A. cryaerophilus* only)
		F87.3	A mix of *Arcobacter thereius* and *Arcobacter cryaerophilus*	Yes (*A. cryaerophilus* only)
88	F88	F88.1	*Arcobacter skirrowii*	Yes
89	M89 (liver)	M89.1	*Arcobacter butzleri*	Yes
101	M101 (liver)	M101.1	*Arcobacter skirrowii*	Yes
		M101.2	*Arcobacter skirrowii*	Yes
		M101.3	*Arcobacter skirrowii*	Yes
		M101.4	*Arcobacter skirrowii*	Yes

F: chicken feces; M: chicken meat; S: chicken cloacal swab.

**Table 5:** BLAST analyses of the amplified *Arcobacter* gene sequences.

Isolate #	Match Description (Accession #)	Percent Similarity	Accession #
F45.1	*Aliarcobacter cibarius* strain LMG 21996 chromosome, complete genome (NZ_CP054051.1)	100	PP937691
F45.2	*Aliarcobacter cibarius* strain LMG 21996 chromosome, complete genome (NZ_CP054051.1)	100	PP937692
F87.1	*Aliarcobacter skirrowii* CCUG 10374 chromosome, complete genome (NZ_CP032099.1)	100	PP937543
F87.2	*Aliarcobacter cryaerophilus ATCC 43158 chromosome*, *complete genome* (NZ_CP032823.1)	100	PP937576
F87.3	*Aliarcobacter cryaerophilus* ATCC 43158 chromosome, complete genome (NZ_CP032823.1)	100	PP937577
F88.1	*Aliarcobacter skirrowii* CCUG 10374 chromosome, complete genome (NZ_CP032099.1)	100	PP937544
M77.1	*Aliarcobacter butzleri* RM4018, complete sequence (NC_009850.1)	100	PP937181
M80.1	*Aliarcobacter butzleri* RM4018, complete sequence (NC_009850.1)	100	PP937182
M84.1	*Aliarcobacter butzleri* RM4018, complete sequence (NC_009850.1)	100	PP937183
M84.2	*Aliarcobacter butzleri* RM4018, complete sequence (NC_009850.1)	100	PP937184
M86.1	*Aliarcobacter butzleri* RM4018, complete sequence (NC_009850.1)	100	PP937185
M89.1	*Aliarcobacter butzleri* RM4018, complete sequence (NC_009850.1)	100	PP937186
M101.1	*Aliarcobacter skirrowii* CCUG 10374 chromosome, complete genome (NZ_CP032099.1)	100	PP937545
M101.2	*Aliarcobacter skirrowii* CCUG 10374 chromosome, complete genome (NZ_CP032099.1)	100	PP937546
M101.3	*Aliarcobacter skirrowii* CCUG 10374 chromosome, complete genome (NZ_CP032099.1)	100	PP937547
M101.4	*Aliarcobacter skirrowii* CCUG 10374 chromosome, complete genome *(NZ_CP032099.1)*	100	PP937548
S84.1	*Aliarcobacter cryaerophilus* ATCC 43158 chromosome, complete genome (NZ_CP032823.1)	96.73	PP943113
S84.2	*Aliarcobacter cryaerophilus* ATCC 43158 chromosome, complete genome (NZ_CP032823.1)	96.77	PP943114

F: chicken feces; M: chicken meat; S: chicken cloacal swab.

## Data Availability

The new nucleic acid sequences have been deposited in the database of GenBank. Accession numbers provided by GenBank ([Internet]. Bethesda (MD): National Library of Medicine (US), National Center for Biotechnology Information; [1982]—[cited 23 June 2024]. Available from: https://www.ncbi.nlm.nih.gov/nucleotide/) have been included in the manuscript in Table 5.

## Abbreviations

The following abbreviations are used in this manuscript:

ATCC	American-Type Culture Collection
BLAST	Basic Local Alignment Search Tool
ERIC	Enterobacterial Repetitive Intergenic Consensus
gDNA	Genomic Deoxyribonucleic Acid
HB	Houf broth
ICMSF	International Commission on Microbiological Specifications for Foods
NCBI	National Center for Biotechnology Information
NRJ-M	Nguyen–Restaino–Juárez-arcobacter chromogenic agar media plates
PCR	Polymerase chain reaction
SGU	Saint George’s University
UPGAM	Unweighted pair group linkage analysis method
